# Pseudohypoparathyroidism, an often delayed diagnosis: a case series

**DOI:** 10.1186/1757-1626-2-6734

**Published:** 2009-05-28

**Authors:** Valentina Donghi, Stefano Mora, Ilaria Zamproni, Giuseppe Chiumello, Giovanna Weber

**Affiliations:** 1 Department of Pediatrics, Vita-Salute San Raffaele UniversityVia Olgettina 60, 20132 MilanItaly; 2 Laboratory of Pediatric Endocrinology, BoNetwork, San Raffaele Scientific InstituteVia Olgettina 60, 20132 MilanItaly

## Abstract

Pseudohypoparathyroidism refers to a heterogeneous group of disorders characterized by parathyroid hormone (PTH) resistance. Pseudohypoparathyroidism is an uncommon sporadic or inherited genetic disorder subdivided into several distinct entities (type Ia, Ib, Ic, type II). We report cases of four children (aged 8 to 13 years) in the winter season 2007-'08. The present work highlights the variable mode of presentation of pseudohypoparathyroidism and the difficulty of an early diagnosis. We stress the importance of a complete biochemical investigation of the calcium-phosphate metabolism to recognize typical biochemical alterations associated with this condition (hypocalcaemia, hyperphosphataemia with increased phosphate tubular reabsorption and elevated PTH levels) in spite of a phenotypic aspect that often lacks the presence of all the peculiar clinical features of Albright hereditary osteodistrophy.

## Introduction

Pseudohypoparathyroidism (PHP) refers to a heterogeneous group of disorders characterized by parathyroid hormone (PTH) resistance. PHP is an uncommon sporadic or inherited genetic disorder subdivided into several distinct entities.

In the past the diagnosis of PHP was confirmed by the administration of bovine or synthetic PTH. This test helped to distinguish patients with PHP type I (characterized by a reduced urinary cAMP and phosphate excretion) from patients with PHP type II (characterized by a normal urinary cAMP excretion and a reduced phosphate excretion). PHP type I is generally classified by subtypes Ia, Ib and Ic, according to different phenotypes, genetic findings and pathogenesis. All subtypes are caused by mutation or imprinting abnormalities in the stimulatory G protein (G_s_). The alpha-subunit of the G_s_(G_s_α) is a signalling protein essential for the actions of PTH and many other hormones.

Type Ia PHP is characterized by resistance to PTH and other hormones that stimulate adenyl cyclase in their target tissues, such as thyroid stimulating hormone (TSH), gonadotropins and growth hormone releasing hormone (GHRH). This hormonal resistance leads to hypocalcaemia, hyperphosphataemia, elevated PTH levels, thyroid and gonadal dysfunction. In addition, type Ia PHP is associated with a constellation of peculiar clinical features collectively termed Albright hereditary osteodystrophy (AHO). These features include short stature, rounded face, brachydactyly, brachymetacarpia, centripetal obesity, subcutaneous ossifications, and in some cases, mental or developmental delay. Patients with type Ia PHP show only about 50% activity of G_s_α subunit. [1-5]

Type Ib PHP is characterized by resistance to PTH mainly in the renal tissue and in a few others tissues such as the thyroid, but without features of AHO [6-8].

Type Ic PHP is the rarest subtype and it is characterized by AHO, resistance to PTH and other hormones in face of normal G_s_α activity.

Patients with PHP type II have hypocalcaemia, hyperphosphataemia and increased serum PTH, but they lack the physical features associated with AHO [9,10].

Pseudohypoparathyroidism is a rare disease and its prevalence in diverse populations is currently unknown. There are only two studies that reported epidemiologic data on pseudohypoparathyroidism: a nation-wide survey carried out in Japan reported a total of 220 cases of PHP between 1968-1977 [11]. More recently another Japanese study reported a prevalence of 3.4 per million population [12].

The aim of our report was to describe the diversity of the clinical presentation and disease course in four children with PHP, observed in our pediatric Unit in the winter season 2007-'08.

## Case presentation

### Patient 1

This case describes a Caucasian 13-year-old boy who presented motor difficulties from birth. He came to our attention after an episode of hypertonia, fixed gaze lasting 1-2 minutes and loss of consciousness. Biochemical analysis, performed during hospitalization in the nearest hospital, showed low calcium concentration (1.1 mmol/l = 4.4 mg/dl) for which he was treated with calcium gluconate 10% ev. He also had elevated PTH concentration (177 pg/ml, reference value 17-73) and hyperphosphataemia (3.16-3.26 mmol/l = 9.5-9.8 mg/dl). The ECG showed a long Q-T interval.

On his admission to our paediatric unit he was in a good general condition, alert and collaborating, orientated in space and time. He did not show signs of tetany. Clinically he appeared tall, with tapering fingers, with neither teeth diastasis nor enamel alterations. He showed mild dysmorphic facial traits, bull neck, kyphosys, barrel-shaped thorax, pseudogynecomasty, but neither rounded face nor brachydactyly, or brachymetacarpia. The biochemical analyses confirmed the hypocalcaemia, hyperphosphataemia and the elevated concentration of PTH with low-normal vitamin D metabolites serum concentration (25OHD: 17.1 ng/ml, reference value 10-68; 1,25(OH)_2_D: 25.1 pg/ml, reference value 15-50) and renal function. There were no other endocrine defects (IGF-I, gonadotropin, testosterone, prolactin, cortisol, adrenocorticotropic hormone were all within the reference range). During hospitalization the calcium gluconate infusion was continued and then converted to oral calcium carbonate (2 g/day) associated with oral calcitriol (1.25 mcg/day).

During the following months several therapeutic adjustments were needed to maintain an adequate calcium-phosphate balance. Currently the patient has achieved a satisfactory calcium-phosphate homeostasis with 1.5 mcg/day of calcitriol.

### Patient 2

A Caucasian 10-year-old boy initially came to our attention for rapidly progressing early puberty. After clinical examination and biochemical hormonal analysis the diagnosis was compatible with a heterozygous deficit of 21-hydroxylase.

Six months later he presented an episode of tonic-clonic seizures associated with loss of consciousness, overflow of saliva, fixed gaze to the right, resolved spontaneously after few seconds. For this reason a computed tomography (CT) scan and a Magnetic Resonance Imaging (MRI) scan of the brain were performed together with an Electroencephalogram (EEG) (basal and after sleep deprivation): all these investigations were normal.

At the age of 11 he referred to the emergency room of our hospital with a lipothymic episode that occurred while playing. Several biochemical examinations were performed: a severe hypocalcaemia (1.75 mmol/l = 7 mg/dl) was detected. Considering the previous convulsive episodes, the boy was hospitalized for further analyses. The calcium-phosphate metabolism revealed a low-normal calcium serum concentration (calcium 2.05 mmol/l = 8.2 mg/dl) and a phosphataemia at the upper normal limits (2.14 mmol/l = 6.42 mg/dl) with a moderately elevated PTH (77 pg/ml, reference value: 17.3-72.9). The thyroid ultrasound scan was normal. At the clinical examination the patient showed a brachymetacarpia of the fourth finger in both hands (this trait was shared by his mother and his grandfather). He did not have any other dysmorphic trait. He began an oral therapy with calcium carbonate (0.5 g/day) and calcitriol (0.25 mcg/day).

A month later his calcium-phosphate metabolism was partially normalized with a phosphataemia still at the upper limits (2.3 mmol/l = 7 mg/dl). In the following months the phosphataemia progressively lowered to the normal values and the patient did not present other convulsive episodes.

### Patient 3

A Caucasian 12-year-old boy came to our attention with a history of two syncopal episodes. The first one had occurred four months before while cycling (the physical stress was associated with an emotional stress because of the bursting of a firecracker). During the following hospitalization the electrocardiogram (ECG) showed a long Q-T interval and the biochemical analyses revealed a mild hypocalcaemia. The echocardiogram and all the ergometric tests were normal. He began a therapy with Nadolol 40 mg/day and the molecular analyses for the long QT syndrome (LQTS) were performed. The cerebral CT scan and the neurological examination were normal. The EEG showed minimal non specific dysfunctional notes.

Persistent hypocalcaemia (2.1 mmol/l = 8.4 mg/dl) with high phosphate serum concentration (2.6 mmol/l = 7.8 mg/dl) was still present a month later.

A second syncopal episode occurred. This time it was preceded by dizziness and coincided with head rotation to the left and hand twitch contractions. During hospitalization the biochemical analyses showed alterations typical of pseudohypoparathyroidism: hypocalcaemia (1.9 mmol/l = 7.6 mg/dl), hyperphosphataemia (3.16 mmol/l = 9.5 mg/dl) and elevated PTH levels (1157 pg/ml, reference values 12-72). The EEG confirmed the non specific disfunction pathway. He started an oral calcium supplementation with calcium carbonate 1 g/day.

This patient finally came to our Institute after a month of calcium treatment. At the clinical examination, signs of latent tetany (Trousseau and Chvostek signs) were present and he had a mild kyphosis attitude. The boy did not show the peculiar clinical features of Albright hereditary osteodistrophy. He began a therapy with calcitriol 0.25 mcg twice a day and calcium carbonate (1 g/day divided in four doses). Ten days later the calcium was slightly higher while the phosphate value did not change. With several therapeutic adjustments the phosphataemia progressively normalized in the following three months and the latest biochemical analyses, performed 6 months after the beginning of the combined therapy (Calcitriol + Calcium Carbonate), showed a normal calcium, a slightly high phosphate, but reduced if compared to the previous value, and a PTH that decreased from 1088 to 162 pg/ml.

### Patient 4

A Caucasian 8-year-old girl came to our outpatient clinics presenting as markedly overweight and with the presence of premature thelarche. Her height was 121.2 cm (3^rd^-10^th^ centile) and her weight 39 kg (>90^th^ centile) with a BMI of 26.7 (>97^th^ centile). She had stretch marks at her hips, acanthosis nigricans on her neck and chest and cellulite. The mammary glands had a diameter of approximately 1 cm. This precocious puberty associated with skeletal abnormalities (she had a marked hypoplasia of the third, fourth and fifth metacarpals shortening and a dysmorphia of the carpal nucleus with a reduced carpal space) found in a radiograph of the left hand and wrist had raised the suspicion of an Albright hereditary osteodistrophy. Several biochemical examinations were performed: all results were within the normal range, except for an elevated PTH (127 pg/ml, reference value 17.3-72.9). The calcium phosphate metabolism, the phenotypic aspect (rounded face, short stature, bull neck, squat hands and feet, epicanthus) and the brachymetacarpia strengthened the diagnostic hypothesis of an Albright hereditary osteodistrophy. For this reason a genetic analysis was planned (still in course).

During the following months she repeated the biochemical analyses, which confirmed normal calcium and phosphate levels, normal 25OHvitamin D and elevated PTH. Two tests (arginine and dexamethasone) were compatible with a growth hormone deficit. The phosphate renal transport (TRP) and the phosphate maximum transport (TmPO4) were elevated.

Table 1 summarizes the biochemical values showed by the four patients at arrival to our Clinic.

**Table 1.** Biochemical values at the arrival to our observation

Refererence Value	Patient 1	Patient 2	Patient 3	Patient 4
Calcium				
2.2-2.7 (mmol/l)	1.26	2.18	1.72	2.32
8.8-10.8 (mg/dl)	5.04	8.72	6.9	9.28
Phosphate				
0.95-1.75 (mmol/l)	2.68	2.14	3.0	1.6
2.85-5.25 (mg/dl)	8.04	6.42	9.0	4.8
PTH				
17.3-72.9 (pg/ml)	219	77	1029	127

## Discussion

PHP is a complex disorder with extreme individual variability. The diagnosis of this rare condition is often delayed, leading to an initially inappropriate approach and therapy. The present work highlights the variable mode of presentation of PHP and the difficulty of an early diagnosis.

In our patients the typical biochemical alterations did not appear in the first years of life, but after the tenth year [2]. Although the initial clinical onset may be related to a severe hypocalcaemia leading to seizures or syncopal episodes, hypocalcaemic condition may become clinically evident only during the pubertal growth spurt, when the calcium requirements are higher. Another important factor to be considered is the seasonal period. It is usually during autumn or winter, when the sun exposure is lower, that the 25OHvitamin D reaches the lowest blood concentration that might lead to hypocalcaemia.

The primary aim of this report is to stress the pivotal role of a complete biochemical investigation of the calcium-phosphate metabolism in every child presenting symptoms suggestive of hypocalcaemia. It is extremely important to perform a complete biochemical analysis (that always includes calcium, phosphate, magnesium and parathyroid hormone serum measurements) in order to distinguish PHP from other disorders. For example, patient 3, after syncopal episodes, performed biochemical analyses that included serum calcium, but not phosphate or PTH, thus preventing a prompt and correct diagnosis. It was only during the second hospitalization that a complete biochemical analysis was made, showing the typical alterations of PHP.

The diagnosis is made more difficult by the various phenotypic aspects correlated with PHP. PHP type Ia and Ic are usually associated with a constellation of peculiar clinical features collectively termed Albright hereditary osteodistrophy (AHO). But the presence of all these phenotypic aspects is rare, and two of our patients (patient 2 and 4) showed only a characteristic brachymetacarpia without any other sign. In contrast, patient 1 did not present any of the typical clinical features of AHO, even if he had some peculiar phenotypic traits (mild dysmorphic facial traits, tallness, tapering fingers, pseudogynecomasty, kyphosys, bull neck, barrel-shaped thorax).

The diagnosis of PHP is nowadays based on clinical and biochemical findings (hypocalcaemia, hyperphosphatemia with increased phosphate tubular reabsorption and elevated PTH levels in subjects having a normal renal function). Molecular characterization is currently a reliable method to differentiate the various subtypes of PHP. Table 2 summarizes the differences between PHPI subtypes. The two main subtypes of PHP, PHP types Ia and Ib, are caused by mutations of *GNAS1*, a gene encoding the alpha subunit of the G stimulatory protein, coupled to the PTH receptor. These gene mutations result in the G protein's inability to activate adenyl cyclase upon the binding of PTH to its receptor. Activation of adenyl cyclase is required for signal transduction that produces the end-organ response to PTH. Failure of signal transduction results in the unresponsiveness of the end organ [8]. *GNAS1* is imprinted in humans so that expression of the allele for a specific tissue is dependent on whether the allele is maternally or paternally inherited. Thus, maternal or paternal transmission leads to different disease manifestations. Heterozygous loss of function mutations in the Gsα gene inherited from the mother lead to PHP Ia, whereas the same mutation inherited from the father leads to pseudoPHP, a distinct entity characterized by AHO, without evidence of hormone resistance. In the thyroid, gonads and pituitary glands, Gsα transcription mainly derives from the maternal allele, thus explaining why only patients with maternally inherited mutations (PHP Ia) show resistance to various other G-protein coupled hormones including TSH, gonadotropins and GHRH [4]. Patients with PHP Ib do not show AHO, and hormone resistance appears to be limited to the renal actions of PTH and occasionally TSH [6,8]. PHP Ib is transmitted only from the mother. There are two variants of PHP type Ib: a familial autosomal dominant form (AD-PHP Ib) and a sporadic form. The proposed mechanism of both forms is the disruption of long-range imprinting control elements of *GNAS1* locus, with consequent decreased Gsα transcription in the proximal renal tubules and PTH resistance [8]. Normally a renal tissue-specific repressor binds to a negative regulatory element on exon 1A and suppresses Gs expression on the paternal allele, but is unable to bind the maternal allele due to methylation, allowing Gsα to be expressed from the maternal allele. In PHP Ib, the methylation of this exon is absent in the maternal allele, allowing the repressor to link to both alleles and suppress the Gsα expression [13].

The aim of PHP therapy is to obtain an adequate calcium-phosphate control and to correct the multiple hormonal resistance, when present. Treatment includes the use of vitamin D active metabolites (alfacalcidol and calcitriol, 20-50 ng/kg/day given in two doses) and calcium supplementation (intravenous calcium to correct symptomatic hypocalcaemia and then oral calcium administration according to individual response and dietary calcium intake). The goal is to maintain blood calcium between 2.2-2.7 mmol/l (= 8.8-10.8 mg/dl), urinary calcium excretion <4 mg/kg/day, and the urinary calcium/urinary creatinine ratio <0.2. For this reason we recommend that all patients undergo a biochemical (calcium, phosphorus, PTH, creatinine) and urinary (calcium and creatinine) examination every three months. A strict follow up is essential to adjust the therapeutic dosage and to preserve a difficult biochemical balance.

**Table 2.** PHP I subtypes can be distinguished by phenotypic traits (AHO*), hormonal resistance, and molecular characterization

	AHO	Hormonal resistance	Molecular characterization (OMIM reference)
PHPIa	Yes	Multiple	#103580
PHPIb	No	Only to PTH	#603233
PHPIc	Yes	Multiple	#103580

*AHO: Albright hereditary osteodystrophy.
